# From signals to systems: the epigenetic-microbiome-mitochondrial axis in IBD pathogenesis

**DOI:** 10.1080/19490976.2026.2692755

**Published:** 2026-07-16

**Authors:** Nesa Kazemifard, Shabnam Shahrokh, Georges Dimitrov, Mehdi Totonchi, Stefan Dimitrov

**Affiliations:** a Research Institute for Gastroenterology and Liver Diseases (RIGLD), Shahid Beheshti University of Medical Sciences, Tehran, Iran; b Pediatrics and Pediatric Surgery, University Hospital Centre of Orléans, Orléans, France; c Laboratory of Immuno-Neuro Modulation (INEM), UMR7355, CNRS and University of Orleans, Orleans, France; d Department of Genetics, Reproductive Biomedicine Research Center, Royan Institute for Reproductive Biomedicine, ACECR, Tehran, Iran; e Institute of Molecular Biology Roumen Tsanev, Bulgarian Academy of Sciences, Sofia, Bulgaria; f Institute for Advanced Biosciences, Inserm U 1209, CNRS UMR 5309, Université Grenoble Alpes, Grenoble, France

**Keywords:** Epigenetic, IBD, microbiome, inflammation, mitochondria, precision medicine

## Abstract

Inflammatory bowel disease (IBD), including Crohn’s disease and ulcerative colitis, is increasingly recognized not merely as an immune-mediated disorder, but as a systems-level condition arising from dynamic interactions among host genetics, environmental exposures, the gut microbiome, and epigenetic regulation. While genetic susceptibility confers risk, accumulating evidence indicates that epigenetic mechanisms act as molecular integrators that translate environmental and microbial signals into sustained transcriptional programs governing immune tolerance, epithelial integrity and tissue repair. Concurrently, intestinal dysbiosis, characterized by loss of short-chain fatty acid-producing commensals and expansion of pro-inflammatory taxa, reshapes host metabolism and chromatin states through microbial-derived metabolites including short-chain fatty acids, secondary bile acids, and tryptophan catabolites. These metabolites affect epigenetic enzymes and modulate the epigenetic chromatin landscape as well as mitochondrial bioenergetics, linking microbial ecology to inflammatory gene regulation. In turn, epigenetic alterations in epithelial and immune compartments influence antimicrobial defense, barrier function, and cytokine networks, thereby sculpting microbial community organization. This bidirectional microbiome-epigenome dialogue creates self-reinforcing circuits that can either sustain mucosal homeostasis or drive chronic inflammation and colitis-associated tumorigenesis. In this review, we synthesize emerging insights into the microbiome–epigenome–mitochondrial axis in IBD and propose a conceptual framework in which metabolic, microbial, and genome-mediated signals converge to determine disease trajectory. We discuss how this integrative perspective may assist biomarker discovery and therapeutic innovation, including epigenetic modulators and microbiota-targeted interventions. Understanding IBD as a dynamically regulated host–microbe ecosystem may accelerate the development of precision strategies aimed at restoring resilient mucosal equilibrium.

## 1. Introduction

Inflammatory bowel disease (IBD) encompasses a spectrum of chronic, relapsing inflammatory conditions of the gastrointestinal (GI) tract, primarily including ulcerative colitis (UC) and Crohn’s disease (CD). UC is confined to the colonic mucosa, whereas CD may affect any part of the GI tract and is characterized by transmural inflammation. The global incidence and prevalence of IBD have increased markedly over the past few decades, particularly in industrialized nations and rapidly developing regions undergoing westernization, posing growing socioeconomic and healthcare challenges.^1-3^ The etiology of IBD still remains unknown, though it is widely accepted to arise from a complex interplay of genetic predisposition, environmental triggers, dysregulated immune responses, and gut microbiome alterations.^4^ ^,^ ^5^ Genome-wide association studies (GWAS) have uncovered over 240 loci associated with IBD risk, many of which localize to non-coding regions involved in transcriptional regulation of immune-regulatory genes.^6^ Despite these advances, genetic factors^7^ ^,^ ^8^ alone cannot account for disease onset as evidenced by the modest concordance rates in monozygotic twins, suggesting a pivotal role for non-genetic modifiers in disease manifestation.^9^

Among these non-genetic contributors, the gut microbiome has emerged as a central player in IBD pathogenesis. Patients with active disease frequently exhibit dysbiosis, characterized by reduced microbial diversity, depletion of beneficial commensals such as *Faecalibacterium prausnitzii*, and expansion of pro-inflammatory taxa including adherent-invasive *Escherichia coli* (AIEC) and *Fusobacterium nucleatum* ^10^. Such microbial shifts have been shown to compromise epithelial barrier function, disrupt metabolic and immunological pathways, and drive mucosal inflammation.^11^ However, the molecular mechanisms linking microbial composition to host immune and epithelial responses remain inadequately understood.

Epigenetic regulation has recently gained prominence as a dynamic interface linking environmental stimuli, including microbiota-derived signals, to stable changes in gene expression.^12^ Epigenetic mechanisms modulate both chromatin structure and function, in particular transcriptional activity, without altering the underlying DNA sequence. These modifications are increasingly implicated in immune cell differentiation, epithelial integrity, and the inflammatory response in IBD.^13^ For instance, aberrant DNA methylation profiles have been identified in the intestinal mucosa and immune cells of IBD patients, correlating with disease phenotype and severity.^14^ Histone modifications further influence gene expression patterns involved in barrier function and cytokine production.^15^ Additionally, dysregulated expression of microRNAs (miRNAs) and long non-coding RNAs (lncRNAs) has been observed in inflamed tissues, where they modulate immune signaling and microbial sensing.^16^ ^,^ ^17^

Importantly, there is growing evidence for a bidirectional relationship between the gut microbiome and host epigenetic pattern. Microbial metabolites, most notably short-chain fatty acids (SCFAs) such as butyrate, propionate, and acetate, serve as potent inhibitors of histone deacetylases (HDACs) and influence DNA methylation.^18^ ^,^ ^19^ Butyrate and propionate inhibit class I and IIa HDACs by directly binding to their catalytic zinc-containing active sites, leading to increased histone acetylation (H3K9ac and H3K27ac) at promoter and enhancer regions of genes involved in immune tolerance and epithelial integrity.^20^ This results in a more permissive chromatin configuration and enhanced transcription of anti-inflammatory mediators such as *FOXP3* and *IL-10*.^21^ ^,^ ^22^ In parallel, SCFAs modulate DNA methylation indirectly by altering intracellular acetyl-CoA and S-adenosylmethionine (SAM) availability, thereby influencing the activity of DNA methyltransferases (DNMTs) and ten-eleven translocation (TET) enzymes.^23^ ^,^ ^24^ Butyrate has also been shown to promote extrathymic regulatory T cell (Treg) differentiation through epigenetic stabilization of the *FOXP3* locus via enhanced acetylation and reduced CpG methylation in conserved non-coding sequences.^25^ Additionally, SCFAs can signal through G-protein-coupled receptors such as GPR41, GPR43 and GPR109A, activating downstream pathways (mTOR, AMPK and STAT3) that converge on chromatin-modifying complexes, further reinforcing transcriptional programs essential for mucosal tolerance and repair.^26^ ^,^ ^27^ Thereby, these mechanisms collectively regulate the expression of genes critical for immune homeostasis and mucosal repair.

Conversely, epigenetic alterations in epithelial and immune cells can modulate the expression of antimicrobial peptides (AMPs) and mucin-related genes, thus shaping microbial composition and susceptibility to dysbiosis.^28^ For example, hypermethylation of promoter regions controlling defensin genes (*DEFB1*) or altered histone acetylation at *MUC2* and *REG3G* loci may impair antimicrobial barrier function, while epigenetic repression of pattern-recognition receptors such as TLRs and NOD2 can disturb microbial sensing.^29^ ^,^ ^30^ In intestinal epithelial cells (IECs), changes in chromatin accessibility at genes regulating tight junction proteins further influence barrier permeability, indirectly modifying microbial niche selection and expansion.^30^

While recent reviews have explored microbiome-epigenome interactions in IBD, most have focused on either mechanistic detail of individual pathways or broad descriptions of host-microbe crosstalk. Instead, this review uses a systems-level perspective that integrates molecular, environmental, and developmental dimensions of the microbiome-epigenome axis. We synthesize evidence spanning the epigenetic machinery together with early-life exposures, lifestyle factors, and pollutants to highlight how these layers jointly contribute to IBD susceptibility and progression. This perspective complements existing frameworks by emphasizing the value of a systems-oriented interpretation of host-microbiome-epigenome dynamics.

## 2. The epigenetic-microbiome axis as a dynamic regulatory network

### 2.1. Bidirectional signaling pathways

**Figure 1.**
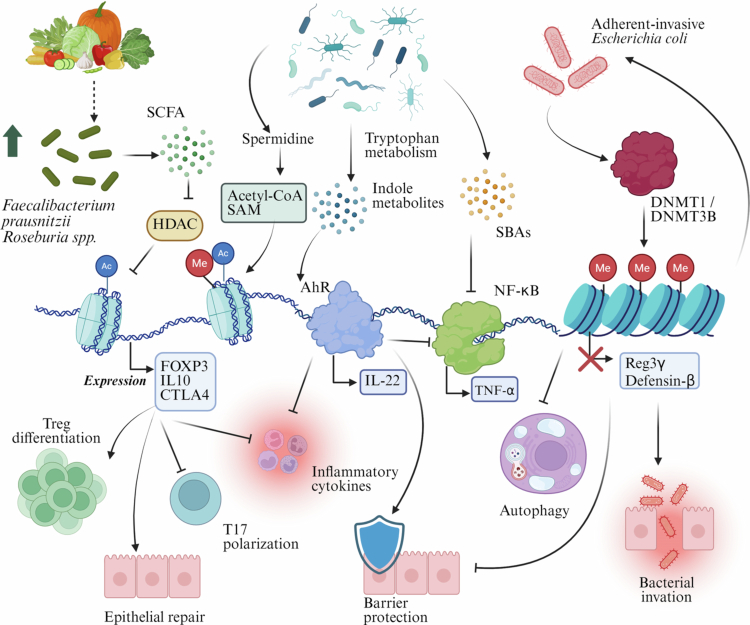
Microbiota-derived metabolites and epigenetic mechanisms regulating intestinal immune homeostasis and inflammation. Dietary fiber fermentation by commensal bacteria, including Faecalibacterium prausnitzii and Roseburia spp., generates short-chain fatty acids (SCFAs), particularly butyrate, which inhibit histone deacetylases (HDACs) and enhance histone acetylation at immunoregulatory loci. Increased acetylation promotes expression of FOXP3, IL10, and CTLA4, supporting Treg differentiation, epithelial repair, and suppression of Th17 polarization and inflammatory cytokine production. Microbial spermidine regulates acetyl-CoA and S-adenosylmethionine (SAM) availability, thereby modulating histone acetylation and methylation dynamics. In parallel, microbiota-derived indole metabolites generated through tryptophan metabolism activate the aryl hydrocarbon receptor (AhR), inducing IL-22 production, promoting epithelial barrier protection, and suppressing NF-κB–mediated inflammatory signaling. Secondary bile acids (SBAs) further contribute to inhibition of NF-κB activation. Conversely, pathogenic Adherent-invasive Escherichia coli induces DNMT1 and DNMT3B expression, leading to promoter hypermethylation and repression of antimicrobial peptide genes, including Reg3γ and Defensin-*β*. These epigenetic alterations impair autophagy and antimicrobial defense, facilitating bacterial invasion and perpetuating intestinal inflammation. Ac, histone acetylation; Me, DNA/histone methylation.

The bidirectional interplay between the gut microbiome and host epigenetic regulation constitutes a central mechanistic layer in IBD pathogenesis. Microbial metabolites reshape host chromatin architecture and transcriptional programs, while host epigenetic states reciprocally regulate antimicrobial defense, barrier integrity, and microbial niche composition (Figure 1). Disruption of this dynamic axis promotes barrier dysfunction, immune dysregulation and consequently, chronic inflammation.

#### 2.1.1. Microbial metabolites as epigenetic modulators

SCFAs generated through fermentation of dietary fibers by commensals such as F. prausnitzii and Roseburia spp.^31^, are among the most extensively characterized microbiota-derived chromatin regulators. Butyrate inhibits class I and selected class II HDACs (except for class III HDACs, class II HDAC6 and HDAC10), enhancing histone acetylation at immunoregulatory loci and promoting transcriptional programs that support Treg differentiation and anti-inflammatory signaling.^32^ ^,^ ^33^ Increased acetylation at the *FOXP3* locus and upregulation of *IL10* and *CTLA4* reinforce mucosal tolerance.^34-36^ In parallel, butyrate suppresses Th17 polarization partly via HDAC3-dependent mechanisms affecting c-Myc-associated metabolic programs.^37^

SCFA signaling through GPR43 and GPR109A further integrates metabolic and transcriptional pathways, biasing differentiation toward regulatory phenotypes and stabilizing epithelial repair programs. Accordingly, depletion of butyrate-producing taxa in IBD correlates with reduced activating histone acetylation at tolerance-associated loci, impaired epithelial bioenergetics, and amplification of NF-κB–driven inflammatory circuits.^31^ ^,^ ^34^ ^,^ ^38^ ^,^ ^39^ These changes establish a feed-forward loop linking dysbiosis to persistent chromatin-mediated inflammatory activation.

Tryptophan-derived indole metabolites represent a second major microbial-epigenetic interface. Indoles activate the aryl hydrocarbon receptor (AhR) in epithelial and immune compartments, promoting barrier-protective transcriptional programs and IL-22–dependent epithelial restitution.^40-42^ AhR signaling also represses NF-κB and HIF1α pathways, restraining glycolysis-dependent inflammatory responses and chemokine production.^43^ ^,^ ^44^ Through coordinated metabolic and transcriptional reprogramming, indole-AhR signaling reinforces epithelial integrity while indirectly shaping microbial composition, forming a regulatory feedback circuit.

Microbial polyamines such as spermidine further illustrate metabolic–epigenetic integration. Rather than directly compacting chromatin, spermidine modulates histone acetylation and methylation dynamics via regulation of acetyl-CoA and SAM availability, influencing epithelial renewal and autophagy-associated transcriptional programs.^45^ ^,^ ^46^ These examples collectively underscore that microbial metabolite function as endogenous chromatin regulators linking luminal ecology to nuclear gene expression.

#### 2.1.2. Microbe-induced DNA methylation and chromatin remodeling

Beyond metabolite-mediated regulation, specific pathobionts directly influence host epigenetic machinery. Adherent-Invasive *Escherichia coli* (AIEC) induces *DNMT1* and *DNMT3B* expression in INCs, promoting promoter hypermethylation of autophagy-related genes and impairing intracellular bacterial clearance.^47^ ^,^ ^48^ This epigenetic silencing compromises “xenophagy” and antimicrobial responses, facilitating intracellular bacterial persistence and perpetuating inflammation.^49^ ^,^ ^50^ Host DNA methylation patterns govern antimicrobial peptides (AMPs) expression and spatial segregation between microbiota and the epithelium. DNA methylation in IECs controls the expression of a cohort of genes, including Reg3γ and Defensin-*β*, which shape microbial niches by selectively eliminating bacterial taxa. These AMPs are secreted into the mucus layer and intestinal lumen, where they bind to bacterial membranes and exert bactericidal or bacteriostatic activity.^51^ ^,^ ^52^ Importantly, DNMT overexpression is not uniform across all IBD patients but appears to be context-dependent and influenced by inflammatory activity and microenvironmental signals. In states of promoter hypermethylation affecting barrier and AMP genes, reduced antimicrobial activity permits closer microbial-epithelial contact and enhanced bacterial penetration. This increases exposure of lamina propria immune cells to microbial-associated molecular patterns (MAMPs), amplifying Toll-like receptor (TLR) signaling and cytokine production, thereby driving chronic immune activation. Thus, epigenetic alterations not only respond to dysbiosis but actively reshape microbial community structure.

Histone modifications provide an additional regulatory layer. Dysregulation of EZH2-mediated H3K27me3 alters repression of inflammatory and regenerative gene programs in IBD, compromising mucosal defense and epithelial renewal.^53^ ^,^ ^54^ In immune cells, epigenetic stability of the *FOXP3* locus remains critical for sustained Treg lineage commitment; reduced acetylation or aberrant methylation at this locus destabilizes tolerance and favors effector-driven inflammation.^55^

#### 2.1.3. Non-coding RNA–mediated host–microbe crosstalk

**Figure 2.**
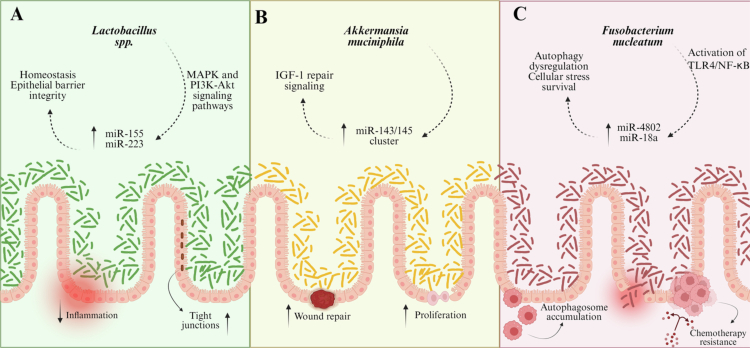
Microbiota-driven regulation of host microRNAs and epithelial responses. (A) Lactobacillus spp.-derived ligands and metabolites activate epithelial and myeloid TLR2/TLR9 signaling and downstream MAPK/PI3K-Akt pathways, leading to NF-κB/AP-1 mediated induction of miR-155 and miR-223, which support epithelial barrier integrity and balanced inflammatory responses. (B) Akkermansia muciniphila induces the miR-143/145 cluster, promoting epithelial restitution and IGF-1–associated repair pathways that facilitate mucosal healing and tissue homeostasis. (C) Fusobacterium nucleatum activates stress-response and pro-survival signaling pathways, including TLR4/NF-κB and β-catenin signaling, resulting in suppression of miR-4802 and miR-18a. Downregulation of these miRNAs contributes to autophagy dysregulation, epithelial stress adaptation, and chemoresistance-associated phenotypes.

Microbiota-driven regulation of non-coding RNAs further refines host-microbe interactions. Commensal bacteria such as *Lactobacillus spp*. modulate miRNAs including *miR-155* and miR-223, influencing epithelial barrier function and inflammatory signaling networks.^56^ Lactobacillus-derived ligands and metabolites can engage epithelial and myeloid pattern-recognition receptors (eTLR2/TLR9) and downstream MAPK/PI3K-Akt pathways, which converge on transcription factors (NF-κB and AP-1) that regulate miRNA transcription^57^ ^,^ ^58^ (Figure 2A). *Akkermansia muciniphila* induces the *miR-143/145* cluster, supporting epithelial restitution and IGF-1–associated repair pathways^59^ (Figure 2B). In contrast, pathogenic Fusobacterium nucleatum alters miRNA expression profiles linked to autophagy dysregulation and epithelial stress responses.^60^ F. nucleatum can activate pro-survival and stress-response signaling in epithelial cells (commonly implicating TLR4/NF-κB and *β*-catenin–associated pathways in the broader literature), which can shift the miRNA landscape toward repression of miRNAs that normally constrain autophagy- and survival-related transcripts. Loss of *miR-4802* and *miR-18a* is therefore proposed to de-repress target mRNAs involved in autophagy control and drug-response pathways, facilitating autophagy imbalance and increased tolerance to cytotoxic stress, features consistent with chemoresistance phenotypes^61^ (Figure 2C).

The microbiota also modulates lncRNA expression. *Bacteroides fragilis* upregulates lncRNA-CGB, leading to enhanced IFN-*γ* signaling.^62^ *CARINH*, a host-derived lncRNA, has been shown to regulate antimicrobial defenses through guanylate-binding proteins (GBPs) illustrating a mechanism by which the host epigenome communicates with and shapes microbial ecology.^63^ A plausible route is that microbial products trigger epithelial/immune sensing pathways that alter lncRNA transcription, after which lncRNA-CGB may act as a scaffold or decoy to regulate the transcriptional machinery controlling IFN-γ–responsive genes.

## 3. Multi-scale effects of epigenetic dysregulation: From cells to systemic inflammation

While genetic studies have identified multiple susceptibility loci, the incomplete heritability of IBD underscores the crucial role of epigenetic reversible modifications changes that regulate gene expression without altering the DNA sequence. Table 1 summarizes key epigenetic alterations implicated in IBD. Epigenetic modifiers alongside with gut microbiome interactions are involved in the regulation of intestinal inflammation, barrier integrity, and immune responses, contributing to disease onset and progression.

### 3.1. Inflammation

Epigenetic remodeling is a defining feature of chronic intestinal inflammation in IBD, linking environmental and immune-derived signals to sustained transcriptional activation of pathogenic gene networks. Among the most reproducible DNA methylation changes is differential methylation at the *VMP1*/*MIR21* locus, encompassing the autophagy regulator *VMP1* (a transmembrane protein crucial for autophagy, ER-membrane contact, and cellular stress responses) and intragenic *miR-21*. Methylation at this region correlates with systemic inflammatory burden and clinical activity indices such as CRP, suggesting that cytokine-driven immune activation reshapes methylation landscapes at inflammation-responsive loci.^64^ ^,^ ^65^ Although the directionality of methylation varies across tissues and cell types, alterations at this locus consistently associate with active disease states.

*TIFAB* is frequently hypermethylated in both CD and UC, implicating epigenetic silencing of negative regulators of inflammation in disease pathogenesis.^66^ ^,^ ^67^ *TIFAB* normally restrains NF-κB signaling upstream of the IKK complex through modulation of innate adapter pathways, including TIFA/TRAF6 interactions.^68^ Its epigenetic repression may therefore lower the threshold for sustained NF-κB activation, a central transcriptional driver of mucosal inflammation.

Beyond DNA methylation, histone modifications critically shape inflammatory transcriptional programs. NF-κB activity is reinforced by acetylation of the p65 (RelA) subunit and by recruitment of histone acetyltransferases such as CBP/p300 to target loci, increasing activating marks including H3K27ac and H3K9ac at cytokine gene enhancers.^69^ ^,^ ^70^ In IBD mucosa, aberrant histone acetylation associates with enhanced expression of pro-inflammatory mediators such as *IL23* and *IFNG*.^71^ ^,^ ^72^ These chromatin states are further stabilized by stimulus-responsive complexes, including HDAC3-containing NCoR/SMRT assemblies, which are essential for orchestrating macrophage inflammatory gene programs in response to IL-1 and TLR signaling.^73^ Rather than functioning as a global deacetylase, HDAC3 occupies a central position integrating signal-dependent transcription factor activation with chromatin remodeling, thereby shaping cytokine amplification and monocyte recruitment. Promoters of inflammatory cytokine genes frequently display activating histone marks such as H3K4me3, reflecting transcriptional competence and sustained accessibility. Together, coordinated alterations in DNA methylation and histone acetylation/methylation establish a permissive chromatin landscape that reinforces NF-κB and STAT-dependent transcriptional circuits. These epigenetic feedback mechanisms contribute to the persistence and self-amplification of mucosal inflammation characteristic of IBD.^68^ ^,^ ^74^

### 3.2. Immune response

Epigenetic remodeling critically shapes immune-cell differentiation, activation thresholds, and effector polarization in IBD. By modulating chromatin accessibility at cytokine loci and lineage-defining transcription factors, DNA methylation and histone dynamics bias mucosal immunity toward sustained inflammatory states while destabilizing regulatory tolerance mechanisms.

A central example is epigenetic dysregulation of the *FOXP3* locus, the master regulator of Treg cell identity. Inflammatory cytokine milieus characteristic of active IBD promote DNMT recruitment and limit TET-dependent demethylation at CpG-rich regulatory elements within FOXP3, shifting the locus toward a repressive chromatin configuration.^75^ ^,^ ^76^ Metabolic perturbations accompanying inflammation may further influence methyl donor availability and methylation stability. Epigenetic repression of *FOXP3* reduces transcriptional durability and impairs Treg suppressive function, thereby weakening mucosal tolerance and permitting expansion of effector T cell responses in the lamina propria. This imbalance reinforces cytokine amplification and chronic immune activation.^77^

In contrast, hypomethylation of pro-inflammatory signaling genes such as *RPS6KA2*, a regulator of MAPK pathways, has been observed in IBD.^78^ Reduced methylation at regulatory regions may increase transcriptional responsiveness to inflammatory stimuli, effectively lowering activation thresholds in innate and adaptive immune cells. Such changes likely reflect inflammation-driven epigenetic remodeling rather than uniform enzymatic shifts, and may arise from context-dependent alterations in DNMT occupancy, TET activity, or immune-cell composition.

Histone-modifying enzymes further reinforce inflammatory circuits. HDAC3, particularly within myeloid lineages, functions as a signal-responsive chromatin regulator that calibrates TLR- and IL-1–induced transcriptional programs.^73^ During intestinal inflammation, enhanced HDAC3 activity promotes NLRP3 inflammasome activation and IL-1β–driven responses, sustaining feed-forward inflammatory loops in experimental colitis.^79^

ATP-dependent chromatin remodelers add an additional regulatory layer. Members of the SWI/SNF complex influence enhancer accessibility and lineage specification in immune and epithelial compartments.^80^ Dysregulation of subunits such as SMARCA5 has been associated with epithelial barrier disruption and secondary immune activation.^81^ Similarly, BRG1 (SMARCA4) modulates nucleosome positioning in innate lymphoid cells (ILC3s), shaping transcriptional balance between protective and pro-inflammatory programs and influencing colitis severity.^82^ Although less directly characterized in IBD, INO80-family remodelers, known regulators of chromatin accessibility and genomic stability, may contribute to maladaptive transcriptional licensing under conditions of persistent inflammatory stress.^83^

Collectively, these findings illustrate that immune dysregulation in IBD is not solely driven by aberrant cytokine signaling but is stabilized by coordinated epigenetic remodeling. Altered DNA methylation, histone modification, and chromatin remodeling establish permissive transcriptional states that lower activation thresholds, amplify inflammatory outputs, and erode regulatory control, thereby sustaining chronic mucosal inflammation.

### 3.3. Barrier function

Intestinal barrier integrity is tightly regulated by epigenetic mechanisms that coordinate epithelial adhesion, immune signaling, and regenerative responses. In UC, methylome analyzes comparing severe and mild disease have identified significant hypomethylation at promoters of immune-regulatory genes, including *IL10*, *NLRP3*, *NLRC4*, *SIGLEC5*, *CD86*, and *CLMP*.^84^ These alterations likely reflect inflammation-driven epigenetic remodeling rather than stochastic variation. Cytokine-rich environments characteristic of active disease can modify DNMT occupancy and chromatin states at transcriptionally poised loci, potentially representing a compensatory attempt to enhance immunoregulatory gene expression under inflammatory stress.^85^ Whether such hypomethylation effectively restores mucosal control remains context-dependent.

In contrast, hypermethylation of *CDH1*, encoding E-cadherin, has been consistently associated with barrier dysfunction in IBD.^86^ Epigenetic repression of *CDH1* compromises adherence junction stability, disrupts epithelial polarity, and increases paracellular permeability. The resulting translocation of luminal antigens and microbial products into the lamina propria amplifies innate immune activation and perpetuates inflammatory circuits. Tight epigenetic control of *CDH1* expression therefore appears essential for maintaining epithelial cohesion and appropriate repair responses. Dysregulated expression, whether through repression or maladaptive remodeling, may contribute to pathological epithelial reprogramming during chronic inflammation.^86^ ^,^ ^87^

Histone modifications provide an additional regulatory layer. H3K27me3 catalyzed by EZH2 within the Polycomb Repressive Complex 2 (PRC2), normally constrains genes involved in epithelial differentiation and regeneration. Reduced H3K27me3 enrichment at specific genomic loci, or its aberrant redistribution due to altered Polycomb Repressive Complex 2 (PRC2) recruitment, has been associated with defective mucosal healing in UC and aberrant activation of repair-related transcriptional programs.^88^ ^,^ ^89^ Inflammatory signaling may perturb PRC2 recruitment or EZH2 activity, disrupting the balance between epithelial proliferation and differentiation required for effective barrier restitution. Collectively, these findings indicate that barrier dysfunction in IBD is not solely a consequence of inflammatory injury but is stabilized by coordinated epigenetic alterations. DNA methylation and histone modifications modulate epithelial adhesion, immune sensing, and regenerative capacity, thereby influencing susceptibility to microbial translocation and the persistence of mucosal inflammation.

### 3.4. Cancer promotion

Perturbations in DNA methylation patterns may also drive the progression of chronically inflamed colonic epithelial cells toward colitis-associated colorectal cancer (CAC). One key mechanism involves hypermethylation of the *DAPK* promoter in UC-associated mucosa.^90^ DAPK is a calcium/calmodulin-regulated serine/threonine kinase that functions as a tumor suppressor by promoting apoptosis, autophagy-associated cell death, and affecting cytoskeletal integrity in response to cellular stress and DNA damage. Under physiological conditions, DAPK participates in p53-mediated apoptotic signaling and limits survival of cells harboring genomic instability. Promoter hypermethylation leads to transcriptional silencing of DAPK, impairing apoptosis of damaged epithelial cells and allowing survival and clonal expansion of cells that accumulate inflammation-induced DNA lesions.^91^ ^,^ ^92^ In the context of chronic UC, persistent oxidative stress, nitric oxide production, and inflammatory cytokine exposure increase DNA damage burden. When DAPK-mediated apoptotic surveillance is epigenetically suppressed, cells with unrepaired mutations evade programmed cell death, thereby increasing the probability of neoplastic initiation. This creates a permissive environment for stepwise accumulation of oncogenic alterations and contributes to the development of CAC.^91^

In addition, comprehensive methylome and transcriptome analyzes of CAC samples have revealed that aminopeptidase N/CD13 (*ANPEP), the* Rho GTPase-activating protein *FAM92A1* and the *STK31*serine/threonine kinase exhibit a significant inverse relationship between promoter methylation levels and transcript expression within the colonic mucosa of CAC patients.^93^ Such inverse correlations suggest epigenetic derepressing or silencing mechanisms that directly influence gene dosage in tumor. development. Functionally, these genes have been implicated in regulation of proliferative signaling, cytoskeletal remodeling, and migratory/invasive behavior. For example, ANPEP (aminopeptidase N/CD13) is involved in extracellular matrix remodeling and can facilitate tumor cell invasion and angiogenesis. STK31, a serine/threonine kinase often categorized among cancer-testis–associated genes, has been linked to enhanced cell cycle progression and oncogenic transformation in GI malignancies, while FAM92A1 has been associated with cytoskeletal organization and cellular motility pathways.^94^

Epigenetic activation or silencing of such genes may therefore shift epithelial cells toward a proliferative, migration-prone phenotype, promoting dysplasia and invasive progression in CAC. Importantly, these alterations occur within a background of chronic inflammatory signaling, which continuously reshapes chromatin accessibility and DNA methylation landscapes, thereby accelerating epigenetically mediated tumor evolution.

**Table 1.** Notable epigenetic changes during IBD development.

Gene/locus name	Function in IBD	Modification	Alteration in IBD	Reference
VMP1/MIR21	Regulates autophagy and inflammation via miR-21	DNA methylation	Hypermethylated in active IBD	[78,95]
DNMT3A	Pivotal regulator in intestinal epithelial homeostasis, Modulating the response to tissue damage	DNA methylation	Hypermethylated, Linked to both CD and UC	[96]
LTA/TNF	Structurally related to cytokines of TNF superfamily	DNA methylation	Hypermethylated, Linked to CD	[96]
AHRR	Immune cell activation and gut homeostasis	DNA methylation	Hypermethylated, Linked to UC	[96]
Zbtb7b	Transcription regulator in CD4 + T cells	DNA methylation	Hypomethylated, Linked to UC	[97]
TRAF6	TNF receptor-associated factor	DNA methylation	Hypermethylated, Linked to CD and UC	[98]
TRIM39-RPP2	Unknown	DNA methylation	Hypomethylated, Linked to pediatric UC	[98]
IL-21R	Th1 suppression and Th2, Th17 and Treg activation, Enhances macrophage pro-inflammatory responses	DNA methylation	Hypomethylated, Linked to CD	[99]
IFNG	Modulating cytokine production in the mucosa	DNA methylation	Hypomethylated in Lamina propria T-cells of IBD patients	[100]
RPS6KA2	Involved in MAPK signaling and immune cell activation	DNA methylation	Hypomethylated in monocytes of IBD patients	[78,95]
SOCS3	Negative regulator of cytokine signaling (JAK/STAT pathway)	DNA methylation	Hypermethylated in UC and CAC	[101]
TCERG1L	Transcription elongation regulator; also linked to colorectal cancer	DNA methylation	Hypermethylated in CD	[102]
TAP1/TESPA1/RPTOR	Antigen processing, T-cell signaling, mTOR pathway regulation	DNA methylation	Hypermethylated in IBD; predictive of treatment escalation	[95]
IL-12/IL-23 Pathway	Key regulators of Th1/Th17 immune responses	DNA methylation	Multiple loci show altered methylation in IBD	[103,104]
IL-23R/IL-17 Axis	Promotes pro-inflammatory Th17 cell differentiation	DNA methylation	Hypomethylated	[105]
TFPI2	Inhibits tissue factor pathway; involved in coagulation and inflammation	DNA methylation	Age-dependent hypermethylation in IBD, Risk marker for CAC	[106]
ITGA4	Integrin involved in leukocyte adhesion and migration	DNA methylation	Hypermethylated in IBD and precancerous lesions	[106]
ITGB2	Beta-2 integrin; immune cell adhesion	DNA methylation	Hypermethylated in CD	[78]
TNF, IL6, IL1B	Activates gene transcription by loosening chromatin	H3K27ac	Increased in inflamed mucosa; linked to pro-inflammatory gene expression	[107]
FOXP3, IL10RA	Repressive mark; silences gene expression	H3K27me3	Decreased in epithelial cells; loss of repression of inflammatory genes	[108]
CDH1, CLDN1	Heterochromatin formation; gene silencing	H3K9me3	Reduced in IBD mucosa; associated with chromatin decompaction	[107]
BRG1 (SMARCA4)	ATPase subunit of SWI/SNF complex; regulates transcription and inflammation	Chromatin remodeling	Downregulated in IECs; impairs barrier and anti-inflammatory gene expression	[82]
SMARCA5	epithelial barrier integrity and innate immune activation	Chromatin remodeling	Downregulated in IECs	[81]

(Abbreviations: CAC = Colitis Associated Colorectal Cancer, IECs = Intestinal Epithelial Cells, Treg = Regulatory T cells, Th = T helper, CD = Crohn’s Disease, UC = Ulcerative Colitis).

## 4. Interplay between mitochondria, gut microbiome and epigenome

### 4.1. Mitochondrial dysfunction in IBD

Investigations have begun to uncover how mitochondrial dysfunction contributes to the development of IBD. Historically, UC was described as a disorder characterized by insufficient epithelial energy supply, suggesting impaired mitochondrial oxidative metabolism within colonocytes.^109^ In CD, structural mitochondrial abnormalities were detected by electron microscopy at early stages of inflammation, preceding overt epithelial barrier breakdown.^110^ ^,^ ^111^ Clinical samples from individuals with UC and CD have demonstrated diminished activity of electron transport chain (ETC) complexes located in the inner mitochondrial membrane. This impairment is thought to result from chronic inflammatory exposure (TNFα and IFNγ) and oxidative/nitrosative stress, which can damage ETC components, oxidize mitochondrial lipids (such as cardiolipin), and disrupt mitochondrial membrane potential. Cytokine-driven activation of NF-κB and JAK/STAT pathways may also alter transcription of nuclear-encoded mitochondrial genes, thereby reducing assembly or stability of ETC complexes. Notably, this impairment appears partially reversible with anti-TNFα therapy, implying that TNFα directly or indirectly modulates mitochondrial activity during intestinal inflammation.^112^ ^,^ ^113^ TNFα signaling promotes mitochondrial ROS production, increases nitric oxide synthesis (which can inhibit complex IV), and triggers mitochondrial permeability transition. Anti-TNFα treatment likely alleviates this cytokine-driven metabolic stress, restoring oxidative phosphorylation capacity and improving epithelial bioenergetic resilience.^114^ ^,^ ^115^

A stress response known as the mitochondrial unfolded protein response (mtUPR) becomes activated in epithelial cells affected by IBD. This pathway is initiated when misfolded or unfolded proteins accumulate within the mitochondrial matrix, leading to retrograde signaling to the nucleus and induction of mitochondrial chaperones and proteases aimed at restoring proteostasis.^116^ Persistent mtUPR activation in IBD may reflect ongoing mitochondrial stress, which can influence inflammatory gene expression through retrograde signaling pathways that intersect with NF-κB and other stress-responsive transcription factors.^117^ Moreover, In patients with UC, chronic inflammation drives oxidative stress, leading to elevated levels of mitochondrial DNA (mtDNA) mutations, particularly in those with colitic cancer. This accumulation of mtDNA mutations during injury-repair cycles may heighten genetic instability and colorectal cancer risk.^118^

Furthermore, mtDNA has been detected in the plasma of IBD patients, where it functions as a damage-associated molecular pattern (DAMP).^119^ Extracellular mtDNA resembles bacterial DNA due to its unmethylated CpG motifs and can activate innate immune sensors by TLR9.^120^ While TLR9 is a major receptor recognizing CpG-rich DNA, mtDNA can also engage cytosolic DNA-sensing pathways such as cGAS/STING and, under certain conditions, contribute to NLRP3 inflammasome activation. These pathways amplify type I interferon responses and IL-1β production, thereby linking mitochondrial damage to systemic and mucosal inflammation.^121^ In addition, abnormalities in Paneth cells have been linked to microbial imbalance and marked downregulation of genes involved in oxidative phosphorylation.^122^ Paneth cells are metabolically active secretory cells that require intact mitochondrial respiration for AMP production. Impaired oxidative phosphorylation may reduce ATP availability, alter redox balance, and compromise granule exocytosis, thereby weakening antimicrobial defense and contributing to dysbiosis. These findings collectively suggest that mitochondrial dysfunction within the intestinal epithelium is both a consequence and amplifier of inflammatory stress.^123^

### 4.2. Bidirectional crosstalk: Mitochondria-epigenome and microbiome-mitochondria interactions

Insights into the dynamic communication between mitochondrial and nuclear genomes have revealed an indirect yet significant role of mitochondrial metabolism in shaping epigenetic modifications. Xie et al. (2007) reported that depletion of mtDNA increased DNMT1 expression and was associated with global CpG hypermethylation.^124^ Complementary restriction landmark genomic scanning (RLGS) confirmed that changes in mtDNA copy number can reshape nuclear DNA methylation landscapes.^125^ Based on these findings, Wallace and Fan proposed the bioenergetics-epigenomics model.^126^ In simplified terms, this model suggests that mitochondrial energy metabolism determines the intracellular availability of metabolites required for chromatin modification. Mitochondria generate or regulate levels of key cofactors such as ATP, acetyl-CoA, NAD⁺/NADH, FAD, and *α*-ketoglutarate. Of note, ATP drives phosphorylation of signaling proteins and histone tails, while AcCoA serves as the primary acetyl donor for acetylation of chromatin and signal transduction proteins, thereby modulating nuclear DNA transcription and replication.^127^ NAD⁺ regulates sirtuin deacetylases; and *α*-ketoglutarate is a cofactor for TET DNA demethylases and JmjC-domain histone demethylases. Therefore, mitochondrial dysfunction can alter the activity of these epigenetic enzymes, leading to changes in chromatin accessibility and gene expression. This framework connects cellular bioenergetics directly to nuclear epigenetic regulation.^128^

Available data indicate that gut microbial populations influence mitochondrial function, mitochondrial biogenesis, and mitochondrial ROS production through bioactive metabolites such as SCFAs and secondary bile acids (SBAs).^129^ In a mouse model of systemic mitochondrial dysfunction (iTfamKO), progressive multiorgan failure is accompanied by gut barrier disruption and reduced levels of microbiota-derived butyrate. Remarkably, restoring butyrate via microbiota transfer or tributyrin administration delays disease progression and extends lifespan.^130^ Moreover, secondary bile acids are microbial derivatives of primary bile acids and can signal through receptors such as FXR and TGR5. Activation of these receptors modulates mitochondrial biogenesis, fatty acid oxidation, and oxidative stress responses, partly via PGC-1α dependent pathways.^131^ Under homeostatic conditions, microbial metabolites enhance mitochondrial oxidative metabolism and maintain epithelial energy balance. In contrast, dysbiosis may disrupt mitochondrial integrity, leading to elevated aerobic glycolysis, reduced oxidative phosphorylation, altered lipid metabolism, increased membrane permeability, and resistance to apoptosis.^132^ Excessive microbial-derived inflammatory stimuli increase mitochondrial ROS and activate HIF-1α dependent metabolic reprogramming, favoring glycolysis over oxidative phosphorylation. Mitochondrial membrane depolarization and impaired *β*-oxidation further destabilize epithelial homeostasis and promote inflammatory signaling.^133^

This bidirectional communication is reinforced by regulatory feedback between nuclear and mitochondrial systems. Nuclear epigenetic programs can alter expression of mitochondrial biogenesis regulators and ETC components, while mitochondria generate metabolic substances that influence nuclear chromatin structure and DNA methylation patterns.^134^ Together, this dynamic network integrates microbial signals, mitochondrial metabolism, and epigenetic regulation into a unified axis that shapes inflammatory susceptibility and disease progression in IBD.

## 5. Drivers of microbiome-epigenetic dysregulation

### 5.1. Early-life determinants and developmental programming

The development of the gut microbiome begins at birth with the formation of bacterial communities, including *Bifidobacterium longum*, *E. coli*, and *B. fragilis*. By the time a child reaches 2 y old, the microbiome starts to take on a composition that is more similar to that of adults, characterized by an increase in *Bacteroidetes*, a decrease in *Proteobacteria*, and a relatively stable level of *Firmicutes*.^135^ The early life phase (first three years) is a crucial period in which exposures related to diet, medications, and both personal and broader environmental factors can influence organ and immune development, potentially leading to long-lasting effects on the offspring and impacting the risk of various diseases after an extended latent period. Research indicates that when a developing fetus and young child are exposed to harmful factors, they may experience disruptions in their microbiome, immune system dysregulation, and a heightened risk of diseases later in life.^136^ A Danish cohort study found that children exposed to oral antibiotics in their first five years had a 33% higher risk of developing pediatric-onset IBD, especially CD. This risk increased with repeated and broad-spectrum antibiotic use.^137^ On the other hand, a Swedish cohort study found that exposure to systemic antibiotics during pregnancy, but not infancy, was associated with a significantly increased risk of very early onset IBD through microbiota alterations, independent of familial IBD or gastroenteritis.^138^ ^,^ ^139^

The maternal microbiota significantly influences the development of the infant microbiome. Specific bacterial strains, such as *Bifidobacterium lactis*, *Bacteroides* spp., and *Lactobacillus* spp., are transferred from the mother to the child. Maternal microbial organisms may contribute to modifying the risk of immune-related diseases in the offspring.^140^ Cesarean section delivery produces a distinct early-life gut microbial profile that is different from that observed with vaginal delivery. This delivery method can interfere with the vertical transmission of certain species, such as maternal *Bacteroides* strains, and delay the colonization of *Bifidobacterium* in newborns. Significant reductions of *Bacteroides* species are often replaced by microbiota originating from breast milk, saliva, and skin, or even by opportunistic pathogens like *Enterococcus*, *Enterobacter*, and *Klebsiella* species.^141^ ^,^ ^142^ Although epidemiological evidence suggests that the method of delivery may not affect the risk of IBD,^143^ more research is necessary to explore the long-term implications of these microbial changes. Several determinant factors including maternal smoking, diet, stress, obesity, exercise, and sleep, as well as preterm birth, antibiotic exposure during early infancy, and the method of infant feeding (breastfeeding versus formula feeding), significantly influence the infant gut microbiome, immune system, and the development of IBD.^136^

Prenatal and early life factors play a critical role in shaping the epigenetic landscape of the developing fetus. Notably, cesarean delivery has been associated with modest but measurable changes in DNA methylation at birth, particularly in genes involved in cellular signaling, development, and immune function.^144^ Additionally, maternal deficiencies in essential micronutrients such as folate, choline, and B vitamins as well as maternal obesity, have been shown to disrupt fetal epigenetic programming.^145^ Importantly, maternal metabolic diseases can affect offspring not solely through genetic inheritance, but also via epigenetic mechanisms. Maternal hyperglycemia and hyperlipidemia, for example, impair fetal organogenesis and metabolic tissue functionality, including insulin signaling pathways.^146^ These disruptions are mediated through epigenetic alterations in genes related to insulin sensitivity, lipid metabolism, and inflammatory processes.^147^ Supporting evidence from both human and animal studies has demonstrated altered DNA methylation patterns in tissues such as the placenta, cord blood, and pancreatic islets, which are associated with increased susceptibility to obesity, type 2 diabetes, and cardiovascular disease.^146-148^ Such epigenetic modifications may also contribute to the phenomenon of "missing heritability" observed in complex diseases like IBD, where familial aggregation exists but no definitive genetic polymorphisms or mutations have been identified.

### 5.2. Lifestyle and environmental modifiers

**Figure 3.**
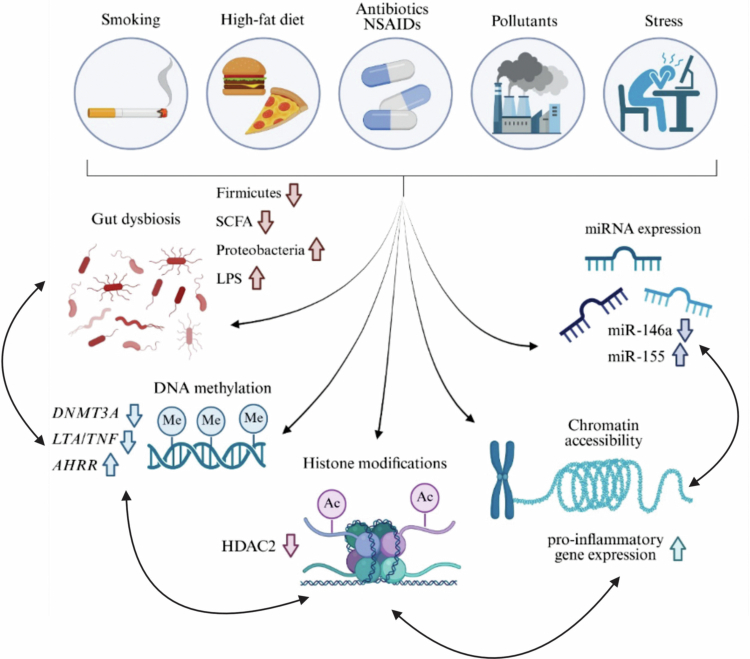
Environmental factors affecting the epigenetic-microbiome axis in IBD. Environmental exposures including cigarette smoking, Western-style high-fat diets, antibiotics, non-steroidal anti-inflammatory drugs (NSAIDs), air pollutants (BPA and PM2.5) and chronic psychological stress, modulate both gut microbiome composition and host epigenetic regulation, thereby contributing to IBD pathogenesis. These factors are associated with gut dysbiosis characterized by increased lipopolysaccharide (LPS) burden, expansion of pro-inflammatory taxa such as Proteobacteria, and depletion of SCFA-producing Firmicutes. Microbial imbalance alters metabolite availability and inflammatory signaling, which in turn affects DNA methylation and histone modification patterns. Environmental triggers have been linked to altered DNA methylation states, including changes in DNMT3A-regulated loci and hypomethylation at inflammatory gene clusters such as the LTA/TNF region, contributing to transcriptional instability and heightened cytokine production. AHRR hypermethylation, particularly in smokers, may impair xenobiotic-response pathways and immune regulatory balance. Cigarette smoke exposure has also been shown to inhibit HDAC2 activity, resulting in increased histone acetylation and sustained activation of inflammatory transcriptional programs. Collectively, these epigenetic alterations modify chromatin organization and transcriptional permissiveness, facilitating inflammatory gene expression and amplifying mucosal immune responses. Environmental stressors further influence non-coding RNA networks, including upregulation of pro-inflammatory miR-155 and downregulation of miR-146a, thereby reinforcing immune activation and impairing resolution of inflammation. It is worth noting that all epigenetic and microbial changes can lead to reciprocal changes in each other (as indicated by the two‑sided arrows), and this requires a systems biology approach to understand the entire complex system.

Environmental exposures are increasingly recognized as key modulators of the gut microbiome and host epigenome, exerting significant influence on intestinal homeostasis and immune regulation. These factors, including diet, lifestyle, pollutants, and pharmaceuticals, interact with microbial composition and metabolic output, which in turn can affect host gene expression through epigenetic modifications. Understanding these bidirectional relationships provides valuable insight into the pathogenesis of IBD and highlights potential intervention points for disease prevention and therapy (Figure 3).

#### 5.2.1. Diet as a primary modulator

Diet is among the most influential environmental factors shaping the gut microbiota and epigenetic regulation. High-fiber diets enhance the abundance of SCFA-producing commensals such as *Faecalibacterium prausnitz*ii and *Roseburia spp*., promoting butyrate production and modulating gene expression linked to anti-inflammatory responses and epithelial barrier function.^32^ ^,^ ^33^ ^,^ ^149^ ^,^ ^150^ Conversely, Western-style diets rich in saturated fats and refined carbohydrates are associated with microbial dysbiosis and reduced SCFA production, leading to decreased histone acetylation and aberrant DNA methylation in epithelial cells. Such dietary patterns correlate with increased intestinal permeability and chronic low-grade inflammation.^151^ Emerging dietary interventions such as the specific carbohydrate diet (SCD) and low FODMAP (fermentable oligosaccharides, disaccharides, monosaccharides, and polyols) diet, have showed potential to modulate microbial composition and restoring SCFA levels. Notably, adherence to the SCD has been associated with clinical remission and histological improvement in pediatric IBD patients.^152^

Polyphenols, bioactive compounds found in plant-based foods such as berries, green tea, and turmeric have been shown to modulate both microbial composition and host epigenetic marks. For example, resveratrol and curcumin can alter histone acetylation and DNA methylation patterns in genes associated with intestinal inflammation, enhancing mucosal immune tolerance and suppressing oxidative stress pathways.^153^ ^,^ ^154^ Moreover, one-carbon metabolism–related nutrients, including methionine, choline, folate, betaine, and vitamins B2, B6, and B12, serve as methyl donors or cofactors in DNA and histone methylation reactions. Imbalances in these nutrients can disrupt methylation homeostasis, potentially influencing susceptibility to IBD and colitis-associated neoplasia.^155^

#### 5.2.2. Lifestyle factors: exercise, stress, and circadian disruption

Physical activity has been linked to favorable shifts in gut microbiota composition including increased diversity and enrichment of SCFA-producing taxa. These microbial changes are associated with elevated SCFA levels and altered histone acetylation, promoting anti-inflammatory gene expression in the colonic mucosa.^156^ In contrast, chronic psychological stress perturbs microbial diversity and composition, increasing the abundance of pro-inflammatory taxa such as *Enterobacteriaceae*. Stress-induced microbial shifts are accompanied by epigenetic alterations in glucocorticoid-regulated genes, leading to impaired barrier function and heightened immune reactivity.^157^ ^,^ ^158^ Moreover, circadian rhythm disruption, often resulting from shift work or irregular sleep patterns, affects microbial diurnal oscillations and epigenetic regulation of host metabolic and immune genes. Misalignment of microbial and host circadian clocks has been associated with increased risk of metabolic inflammation and colitis.^159^ ^,^ ^160^

#### 5.2.3. Environmental pollutants and epigenetic reprogramming

Exposure to environmental toxins including heavy metals, pesticides, and endocrine-disrupting chemicals, has been implicated in gut dysbiosis and epigenetic dysregulation. Bisphenol A (BPA), a widely used plasticizer, alters microbial composition by promoting the growth of *Proteobacteria* and reducing beneficial *Firmicutes*. Simultaneously, BPA exposure induces aberrant DNA methylation in immune-related genes, including *IFN-γ*, enhancing susceptibility to mucosal inflammation.^161-163^ Moreover, airborne particulate matter (PM2.5 and PM10) has been shown to impair gut barrier function and promote colitis through oxidative stress, inflammation, and histone modifications in IECs. Exposure to air pollution correlates with increased incidence of IBD in urban populations.^164^ ^,^ ^165^

#### 5.2.4. Tobacco smoking and the microbiome-epigenome interface

Cigarette smoking alters microbial communities, decreasing *F. prausnitzii* and increasing Clostridium and *Bacteroides-Prevotella* species. These shifts contribute to increased mucosal inflammation and may impair therapeutic response in smokers with CD.^166^ Smoking exerts complex effects on IBD risk and phenotype, increasing susceptibility to CD while paradoxically exerting a protective effect in UC. Recent epigenome-wide association studies (EWAS) have identified smoking-associated DNA methylation changes in genes such as DNMT3A, AHRR, and LTA/TNF, which are implicated in immune regulation and epithelial homeostasis.^167^

#### 5.2.5. Pharmaceuticals and epigenetic-microbial disruption

Pharmacologic agents, particularly antibiotics, nonsteroidal anti-inflammatory drugs (NSAIDs), and proton pump inhibitors (PPIs), can profoundly influence the microbiome-epigenome interface. Broad-spectrum antibiotics reduce microbial diversity, deplete SCFA-producing taxa, and alter epithelial DNA methylation profiles, effects that may persist for months and increase IBD susceptibility.^168^ ^,^ ^169^ NSAIDs exacerbate mucosal injury by affecting prostaglandin synthesis, disrupting barrier function, and influencing histone acetylation patterns in epithelial cells. PPIs have been linked to overgrowth of *Enterobacteriaceae* and altered methylation of genes regulating tight junction integrity.^170-172^

Immunosuppressive agents, including corticosteroids and biologics, modulate host epigenetic responses. Prolonged glucocorticoid use alters methylation of T cell-associated genes and impairs CD4 + effector function, while anti-TNF therapy in CD patients induces specific methylation changes at loci such as *SOCS3*, correlating with therapeutic response.^173^ ^,^ ^174^

## 6. Biomarkers and predictive models: beyond static readout

### 6.1. Longitudinal multi-omics profiling

**Figure 4.**
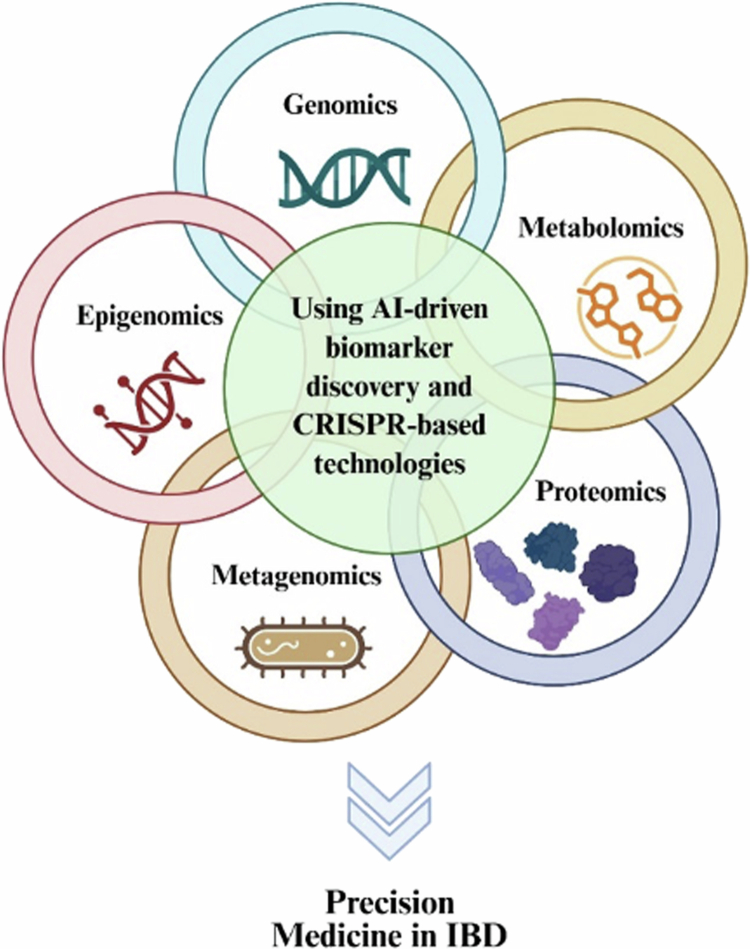
Multi-omics approaches for precision medicine in IBD. Genomic and epigenomic profiling reveals individual genetic predispositions and epigenetic modifications, informing targeted interventions. Metagenomic sequencing of the gut microbiome facilitates microbial characterization, guiding therapies such as probiotics and fecal microbiota transplantation to restore intestinal balance. Proteomic and metabolomic mapping uncovers biomarker signatures and key metabolic pathways, supporting early diagnosis and precision treatment. CRISPR-based technologies allow for precise editing of disease-associated genes, offering promising avenues for long-term disease management. Meanwhile, AI-driven biomarker discovery enhances diagnostic accuracy and therapeutic selection through predictive modeling. Together, these integrative methodologies advance precision medicine in IBD, enabling tailored interventions that improve clinical outcomes.

The complexity of IBD pathophysiology demands systems-level models incorporating multi-dimensional data (Figure 4). Integrating host-microbiome-epigenome interactions through machine learning frameworks can aid in stratifying patients by disease subtype, flare risk, or treatment responsiveness.^175^ Such models may also identify causal regulatory circuits involving microbial metabolites, transcriptional regulators, and chromatin modifiers. Moving forward, the combination of single-cell multi-omics, long-read epigenomics, and AI-driven network inference will be critical for achieving a granular understanding of how microbiome-epigenome interactions operate across spatial and temporal scales in the human gut.^176^ The clinical IBD community holds high hopes that multi-omics studies deliver dependable biomarkers and enable accurate forecasts of remission, relapse, and treatment response.^177^ An optimal set of complementary biomarkers should integrate insights from multiple genetic, microbial, transcriptomic, proteomic, metabolic, and immunological data types through a unified multimodal analysis and achieve a genuinely personalized approach in IBD treatment.^178^

### 6.2. Functional and network-based biomarkers

High-dimensional multi-omics data, which goes beyond analyzing single shifts in the genome, epigenome, or metagenome, can provide deep understanding about the causal pathways involved in IBD and introducing valuable biomarkers using advanced computational methods. Over the past decade, network analysis has emerged as a powerful approach for integrating various omics datasets. This method helps identify key drivers and biological pathways that contribute to the onset and progression of the disease.^179^ Peters et al. ^180^ mapped out an intricate regulatory network linking GWAS loci to functional gene circuits in IBD. By integrating genomic, transcriptomic, and epigenomic data, alongside expression quantitative trait loci (eQTLs) and regulatory elements, they identified 12 key genes that serve as central regulators of inflammation. These central genes not only illuminate disease mechanisms but also correlate strongly with its severity and duration, highlighting their potential as clinical biomarkers.^180^ On the other hand, Ning et al. constructed multi-omics biological correlation (MOBC) maps to link microbial taxa with metabolite profiles. Their study identified three previously underreported bacterial species associated with IBD: *Asaccharobacter celatus*, *Gemmiger formicilis*, and *Erysipelatoclostridium ramosum*, along with 36 significantly altered metabolites. This research contributed to the development of multi-omics diagnostic biomarkers that demonstrate high accuracy across global cohorts.^181^ Moreover, Serrano-Gómez et al.^182^ conducted a multi-omics study on fecal samples from patients with CD. They developed a metagenome-metatranscriptome-metabolome network and utilized machine learning models to predict disease status. The study identified a panel of 20 microbial species that are specific to CD and demonstrate high diagnostic accuracy, along with disrupted microbial fermentation pathways in the gut bacteria. These markers are specific to CD, making them valuable for distinguishing between Crohn's disease and UC.^182^

On this manner a comprehensive analysis examining the correlations between DNA methylation patterns and microbiome composition in pediatric IBD patients has revealed significant age-dependent alterations in DNA methylation within the colonic mucosa and a dynamic process of epigenetic remodeling throughout childhood and adolescence.^183^ The study also highlights notable developmental shifts in the composition and diversity of the mucosal microbiota over time. Collectively, these interconnected changes between the epigenome and microbiome indicate a coordinated maturation process that likely influences disease susceptibility and progression.^183^ Unfortunately, epigenomics is a less investigated omic in IBD, and the systemic studies of the interaction between the epigenome and gut microbiome, or other omics, are more scarce.^184^ Noble et al.^185^ introduced a model in which epigenetic mechanisms mediate gene-environment interactions (GxE), especially during early-life periods of heightened vulnerability. They emphasized the importance of integrating GWAS, epigenetic data, and environmental exposure research including factors like the microbiome, diet, and smoking to inform novel preventive approaches and personalized therapies for IBD.^185^ The data on omics, pathogenesis, and treatment strategies for IBD has rapidly increased, making it an ideal candidate for AI support.

AI can help identify IBD features including the epigenome-microbiome interaction to enhance clinical outcomes. The integration of AI and multi-scale hybrid models is paving the way for digital twins of patients, allowing for precision dosing and personalized treatment.^186^ Digital twins are virtual models of physical systems used to simulate behavior, widely applied in industries like manufacturing. In healthcare, digital patient twins based on personal and population data, offer potential for personalized treatment and real-time monitoring. However, their routine use is limited due to challenges like unreliable drug response simulations, costly individualized drug production, and regulatory hurdles. Overcoming these barriers requires more digital innovation in healthcare.^187^

## 7. Translational challenges: from insight to therapy

### 7.1. Precision epigenetic editing and microbiome modulation

**Figure 5.**
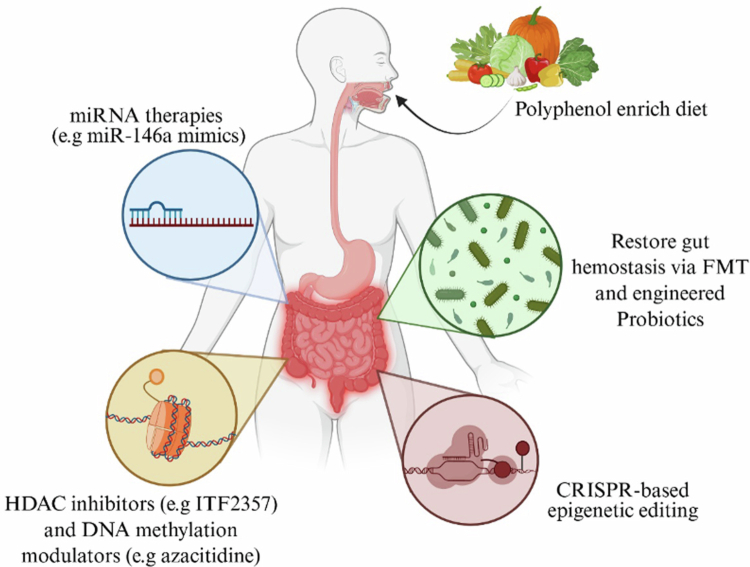
Therapeutic interventions targeting epigenetics and the microbiome. Extensive research into epigenetic alterations and gut microbiome dysbiosis associated with IBD has facilitated the development of targeted therapeutic strategies aimed at restoring intestinal homeostasis and modulating inflammatory responses. Dietary interventions, particularly polyphenol-rich regimens, have shown promise in influencing epigenetic regulation and reshaping microbial composition. Microbiome restoration through fecal microbiota transplantation (FMT) and genetically engineered probiotics supports gut equilibrium and enhances barrier integrity. miRNA-based therapies, such as those mimicking miR-146a, offer potential for immune modulation and inflammation control. Epigenetic modulators (including histone deacetylase inhibitors (butyrate analogs, ITF2357) and DNA methylation agents (azacitidine derivatives)) have demonstrated efficacy in suppressing pro-inflammatory pathways. Additionally, CRISPR-based epigenetic editing introduces a novel platform for precise gene regulation, advancing personalized treatment approaches. Collectively, these innovations highlight the therapeutic potential of integrating epigenetic and microbiome-targeted strategies in IBD management.

Despite significant progress in the management of IBD through biologics, small-molecule inhibitors, and immunomodulators, a considerable proportion of patients remain refractory to current therapies, relapse after remission, or experience adverse effects due to systemic immune suppression. These limitations underscore the need for novel therapeutic approaches that target underlying molecular mechanisms, including the microbiome-epigenome interface, which plays a central role in mucosal inflammation, barrier dysfunction, and immune dysregulation in IBD (Figure 5).

One promising avenue is the modulation of gut microbiota through diet, probiotic supplementation or fecal microbiota transplantation (FMT). Dietary strategies hold significant potential for modulating the gut microbiota in managing IBD. Diets rich in polyphenols help reduce intestinal inflammation and influence macrophage activity.^188^ A daily routine that emphasizes fruits and vegetables paired with moderate intake of oily fish, dairy, and poultry, while minimizing red and processed meats, is linked to a lower risk of chronic diseases in a population study.^189^ Moreover, based on a human randomized trial, low FODMAP diet in patients with quiescent IBD for over four weeks showed significant improvement in abdominal pain, bloating, stool frequency and microbiome composition compared to controls, without exacerbating intestinal inflammation as measured by fecal calprotectin and CRP.^190^ However, It is important to note that current ESPEN guidelines explicitly state that there is no single ‘IBD diet’ that can be generally recommended to induce remission in active IBD.^191^ Therefore, while no universal IBD diet exists, dietary interventions may be considered as adjunctive strategies in selected patient subgroups, pending further high-quality trials.

In IBD, butyrate-producing bacteria are markedly depleted, suggesting that dietary or probiotic/prebiotic/synbiotic-based strategies aimed at restoring these communities may improve mucosal immunity and clinical outcomes.^192^ Engineered probiotic strains are developed to go beyond the benefits of natural probiotics by actively interacting with their environment or the human host in smarter and more targeted ways. For example, engineered AIEC Nissle 1917 (EcN), which is designed to overexpress two antioxidant enzymes, has been shown to reduce intestinal inflammation and restore microbial balance and epithelial barrier function in a murine model of IBD.^193^ In a separate study, engineered EcN was orally administered with Eudragit L100-55 enteric coating to facilitate targeted delivery of IL-2 in the gut.^194^ In a DSS-induced colitis mouse model, this formulation significantly enhanced Treg activation, modulated innate immune responses, rebalanced the gut microbiota, and promoted epithelial barrier repair.^194^ Although preclinical studies on engineered probiotics have shown promising therapeutic potential, human trials have yet to be conducted. Therefore, safety concerns, particularly regarding the use of genetically modified bacterial strains, must be carefully addressed before advancing to clinical applications.

Clinical studies have shown that FMT can restore microbial diversity and induce remission in subsets of UC patients. FMT has also been reported to impact DNA methylation patterns and histone modifications in immune cells, although the exact mechanisms remain to be fully elucidated.^195^ ^,^ ^196^ In a randomized, placebo-controlled trial on active UC patients, FMT showed promising results in improving clinical and endoscopic remission and microbial diversity without increasing adverse events.^197^ However, findings from human trials in CD remain inconsistent. A pilot randomized study reported that FMT group demonstrated higher steroid-free remission rates at 10 and 24 weeks compared to the control group.^198^ Conversely, a separate double-blind, placebo-controlled trial was terminated early due to futility, as no FMT recipients reached the primary endpoint of combined clinical and endoscopic remission at week 8.^199^

On the other hand, CRISPR-based epigenetic editing offers a next-generation strategy to modulate disease-relevant gene expression without altering DNA sequence. Catalytically inactive Cas9 (dCas9) fused to epigenetic effector domains allows for targeted DNA demethylation or histone acetylation at inflammatory gene loci. Although still preclinical, this technology could enable durable, tissue-specific reprogramming of inflammatory responses in IBD.^200^ ^,^ ^201^ Researchers developed an oral CRISPR-Cas9 delivery system to treat IBD by targeting TNF-*α*. The system protects gene-editing nanoclusters through multiple layers (outer membrane vesicles and calcium alginate microspheres) allowing safe passage through the stomach and precise release in the intestines. Once there, the nanoclusters penetrate inflammatory cells and edit TNF-*α* genes, offering a promising therapeutic strategy for IBD.^202^ In another study, a *pH*-responsive nanoparticle system (CCZM) combining a ROS-scavenging nanozyme and CRISPR/Cas9 targeting CD98 for UC showed targeted delivery, immune evasion, and inflammation-specific action. *In vitro* and mouse models that downregulated CD98 and alleviated UC symptoms, offering a precise therapeutic strategy.^203^ These preclinical studies show promise; however, to date, no human or clinical studies using CRISPR technologies in IBD patients have been conducted.

Moreover, epigenetic drugs offer a mechanistic strategy to reverse pathogenic chromatin states in IBD. HDAC inhibitors such as vorinostat and ITF2357 (givinostat) have shown anti-inflammatory properties by reducing NF-κB activity and cytokine production in preclinical models.^15^ ITF2357 decreases inflammation (reduced IFN-*γ*, elevated IL-10), shrinks tumor size and count, and induces apoptosis through histone 3 acetylation and HDAC-dependent NF-κB regulation in mouse colitis (DSS/TNBS) and inflammation-associated colon cancer models (AOM/DSS, IL-10 KO).^204^ In an LPS-stimulated human PBMCs, it reduces pro-inflammatory cytokines (TNFα, IL-1α/β and IFNγ) without apoptosis and downregulates TNFα/IFNγ mRNA.^205^ However, ITF2357 has entered early-phase clinical trial (NCT00792740) for moderate-to-severe CD in 2007 but it Prematurely Ended.^206^

miRNAs are capable of regulating hundreds to thousands of genes, making them powerful master regulators in complex, multi-gene chronic diseases. For instance, miR-146a is notably overexpressed in IBD and plays a protective role in mitigating colitis symptoms. In experimental mouse models of IBD, treatment with miR-146a mimics significantly reduced inflammation and disease severity, highlighting its potential as a promising candidate for RNA-based therapeutic development.^207^ However, no human studies have been initiated for miRNA therapy in IBD due to challenges with delivery and stability.

### 7.2. Barriers to implementation and future directions

While emerging therapies targeting epigenetic regulators and microbial dysbiosis hold promise, many challenges remain in translating preclinical findings into clinically viable treatments (Table 2). Based on several studies, bacterial metabolites have been recognized for their beneficial effects in the context of IBD; however, excessive levels of these metabolites may exert adverse consequences. For instance, elevated concentrations of secondary bile acids have been associated with hepatotoxicity, proliferation of pathogenic bacterial species, heightened risk of colorectal cancer, and an increased likelihood of cardiovascular events.^208^ Furthermore, certain studies have indicated that oral administration of SCFAs alone may not fully achieve their anti-inflammatory and metabolic effects. In some instances, SCFAs have been shown to disrupt gut barrier function through metabolic reprogramming during acute T cell-mediated inflammation. It is evident that the effects of different types of SCFAs may vary, and their biological activities are likely influenced by the health status of the host. Consequently, further high-quality epidemiological studies with larger sample sizes are essential to validate the effects of SCFAs in human populations.^209^

The current landscape of probiotic usage is characterized by a significant lack of standardization concerning dosage, duration, delivery methods, and formulations. Evidence suggests that probiotics generally exhibit efficacy in the management of UC; however, clinical trials have largely demonstrated minimal to no benefit in CD. Consequently, existing clinical guidelines do not endorse the use of probiotics for CD due to insufficient supporting evidence.^191^ ^,^ ^210^ Given the uniqueness of each individual's gut microbiome, a probiotic that proves beneficial for one patient may be ineffective or even deleterious for another. This emphasizes the necessity for personalized probiotic therapy, a field that remains in the nascent stages of development. On the other hand, although EcN is widely used as a chassis for engineered probiotics in disease treatment, its biological safety remains uncertain. A major concern is the reliance on plasmid-based systems, which require continuous selection pressure and pose risks of horizontal gene transfer to other microbes. These artificial genetic elements may unpredictably interact with host systems and cause unintended side effects, presenting a significant barrier to the clinical translation of engineered microbial therapies.^211^

Bacterial extracellular vesicles (BEVs) have emerged as a promising alternative to live probiotics for treating IBD. These nanoscale vesicles, secreted by bacterial cells, are rich in bioactive molecules and exhibit similar immunomodulatory and environmental regulatory functions as live probiotics. However, BEVs offer distinct advantages, including improved stability and reduced safety concerns related to viability.^212^ Despite their potential, the precise mechanisms by which BEVs influence host epigenetic regulation remain largely unexplored, highlighting a critical area for future research.

Major challenges in FMT development in IBD treatment encompass ensuring recipient safety, determining optimal dosing parameters accounting for confounding variables including diet, environmental exposures, ethnicity, comorbidities, and non-antibiotic medications, assessing the durability of clinical responses, and elucidating the influence of recipient-specific factors such as genetics, immune profiles, dietary habits, and baseline microbiome composition. Although stringent donor screening protocols mitigate transmission risks, they do not fully eliminate them. Furthermore, the majority of investigations overlook mucosa-associated and small intestinal microbiota; the deployment of advanced robotic capsules may facilitate comprehensive sampling along the entire GI tract.^213^

The development of HDAC inhibitors and DNA methyltransferase inhibitors for IBD treatment has been hindered by concerns regarding off-target effects and systemic toxicity. The development of inhibitors targeting specific modifications presents considerable challenges, particularly in minimizing interference with other modifications. Achieving specificity in targeting individual enzymes is complex and often risks disrupting other enzymes and associated pathways. Furthermore, addressing the potential off-target effects, variability in metabolic processing, and determining optimal dosing and treatment duration for inhibitor enzymes remain significant obstacles in advancing this therapeutic approach.^214^ Moreover, it is important to acknowledge that epigenetic modifications exhibit dynamic and often transient characteristics, which can result in a lack of stability or permanence. This inherent variability poses significant challenges and limitations for their application in therapeutic interventions, as the durability of such changes may not consistently support long-term efficacy.^215^

CRISPR-based epigenetic editing strategies demonstrate significant potential for personalized therapeutic interventions; however, several critical challenges within this field must be addressed to fully harness its capabilities. Notably, the off-target effects associated with CRISPR-mediated epigenetic modifications can lead to unintended alterations in the expression of non-target genes, necessitating improved specificity and efficacy. Furthermore, the development of highly precise target selection and delivery systems is essential to achieve optimal therapeutic outcomes without triggering undesirable gene expression changes.^215^ ^,^ ^216^ Another pressing concern lies in the immune response elicited by the CRISPR system, which can manifest as allergic or inflammatory reactions that may exacerbate disease progression.^216^ To overcome these barriers, robust methodologies must be developed to mitigate immune activation and ensure the safety of these therapeutic approaches. Addressing these limitations is pivotal for advancing CRISPR-based epigenetic therapies into clinical practice and realizing their full potential in treating complex diseases such as IBD.

**Table 2.** Summary of emerging therapeutic modalities targeting the microbiome–epigenome axis in IBD.

Modality	Evidence level	Clinical data	Key risks	Next step
Dietary change	Observational + small interventional	Diet trials in IBD	Variable adherence, microbiome heterogeneity, lack of standardized protocols	Larger controlled trials, personalized plans
Bacterial metabolites	Preclinical + observational human data	Limited (butyrate studies)	Variable production, delivery challenges, host-microbiome variability, toxicity	Larger controlled trials, formulation refinement, targeted delivery
Probiotics	Clinical trials (UC); limited in CD	Yes	Lack of standardization, strain-specific effects, poor efficacy in CD	Personalized formulations, CD-specific trials
Bacterial EVs	Preclinical	No	Mechanistic uncertainty, delivery optimization, regulatory unknowns	Mechanistic studies, safety profiling
Engineered probiotics	Preclinical	Limited (early-phase)	Colonization efficiency, immune activation, strain-specific effects, risks of horizontal gene transfer	Safety trials, strain optimization
FMT	Clinical studies (mostly in UC)	Yes	Donor variability, infection risk, unclear long-term effects, poor efficacy in CD	Standardization, mechanistic studies
miRNA therapies	Preclinical	No	Off-target gene regulation, delivery stability, immune modulation	Formulation development, phase I trials
HDAC inhibitors	Preclinical	Prematurely terminated phase ΙΙ trial	Systemic toxicity, limited efficacy endpoints, inherent variability, cytokine suppression only, low stability of changes	Targeted delivery, biomarker validation, personalized plans
DNA methylation agents	Preclinical	No	Global demethylation risk, off-target gene activation, delivery limitations, inherent variability	Targeted delivery development, phase I trials, personalized plans
CRISPR-based epigenetic editing	Preclinical	No	Off-target effects, delivery challenges, immunogenicity	Safety profiling, delivery vector refinement

This table outlines the current translational landscape of novel interventions under investigation for IBD. Each modality is evaluated based on its evidence level, availability of human data, key risks, and next steps required for clinical advancement. The table emphasizes the need for cautious interpretation of preclinical findings and highlights the translational gaps that must be addressed to realize clinical impact.

## 8. Toward an integrative IBD paradigm

IBD emerges from a complex interplay between host genetics, environmental exposures, and microbial dysbiosis, with the epigenome serving as a dynamic interface that translates these signals into persistent transcriptional programs. This review highlights how the microbiome-epigenome axis is not only a mechanistic driver of mucosal inflammation and barrier dysfunction but also a promising translational target. We integrated early-life and environmental determinants with molecular epigenetic mechanisms, emphasize the bidirectionality of host-microbe regulation, and discuss their convergence in shaping immune and epithelial responses. Furthermore, we bring forward emerging translational avenues, from CRISPR-based epigenetic editing to engineered probiotics and miRNA mimics, while critically assessing their challenges and limitations.

Looking ahead, the future of IBD research lies in longitudinal, integrative multi-omics frameworks that combine genomic, epigenomic, microbial, and metabolic data with AI-driven models and patient-specific digital twins. Such approaches will allow us to move beyond descriptive associations toward predictive and actionable biomarkers, enabling true precision medicine. By reframing the microbiome-epigenome axis as both a biological interface and a clinical lever, this review underscores its central role in unraveling IBD pathogenesis and guiding the next generation of diagnostics and therapies.

## Abbreviations

5-ASA: 5-Aminosalicylic Acid
2D: Two-Dimensional
3D: Three-Dimensional
AAV: Adeno-Associated Virus
AhR: Aryl Hydrocarbon Receptor
AIEC: Adherent-Invasive Escherichia coli
AIM2: Absent in Melanoma 2
AMP: Antimicrobial Peptide
BEV: Bacterial Extracellular Vesicle
BPA: Bisphenol A
CAC: Colitis-Associated Colorectal Cancer
cGAS–STING: cyclic GMP-AMP Synthase–Stimulator of Interferon Genes
cGMP: cyclic Guanosine Monophosphate
CGD: Chronic Granulomatous Disease
CLDN: Claudin
CM: Central Memory T cells
CRP: C-Reactive Protein
CSD: Congenital Sodium Diarrhea
CVID: Common Variable Immunodeficiency
CD: Crohn’s Disease
DNMT: DNA Methyltransferase
DAMP: Damage-Associated Molecular Pattern
DSS: Dextran Sulfate Sodium
dsDNA: Double-Stranded DNA
DUOX2: Dual Oxidase 2
EDA-ID: Ectodermal Dysplasia with Immunodeficiency
EM: Effector Memory T cells
EO-IBD: Early-Onset Inflammatory Bowel Disease
ER: Endoplasmic Reticulum
ETC: Electron Transport Chain
EWAS: Epigenome-Wide Association Study
FMT: Fecal Microbiota Transplantation
FOXP3: Forkhead Box P3
GI: Gastrointestinal
G6P: Glucose-6-Phosphate
G6PC3: Glucose-6-Phosphatase Catalytic Subunit 3
GOF: Gain-of-Function
GPR: G-Protein-Coupled Receptor
GSD1B: Glycogen Storage Disease Type Ib
GWAS: Genome-Wide Association Studies
HDAC: Histone Deacetylase
HIF-1α: Hypoxia-Inducible Factor-1 Alpha
HLH: Hemophagocytic Lymphohistiocytosis
HNF4A: Hepatocyte Nuclear Factor 4 Alpha
HSCT: Hematopoietic Stem Cell Transplantation
IBD: Inflammatory Bowel Disease
IBDU: Inflammatory Bowel Disease Unclassified
IC: Indeterminate Colitis
IEC: Intestinal Epithelial Cell
IFN: Interferon
IL: Interleukin
INO80: INO80 Chromatin Remodeling Complex
IO-IBD: Infantile-Onset Inflammatory Bowel Disease
ISG: Interferon-Stimulated Gene
ITGA4: Integrin Alpha 4
ITGB2: Integrin Beta 2
KO: Knockout
LRBA: Lipopolysaccharide-Responsive Beige-Like Anchor Protein
lncRNA: Long Non-Coding RNA
LOF: Loss-of-Function
LPS: Lipopolysaccharide
MAPK: Mitogen-Activated Protein Kinase
miRNA: MicroRNA
MOBC: Multi-Omics Biological Correlation
mtDNA: Mitochondrial DNA
mtUPR: Mitochondrial Unfolded Protein Response
NADPH: Nicotinamide Adenine Dinucleotide Phosphate
NF-κB: Nuclear Factor Kappa B
NLRP3: NOD-Like Receptor Family Pyrin Domain-Containing 3
NSAID: Non-Steroidal Anti-Inflammatory Drug
PDOTS: Patient-Derived Organotypic Tumor Spheroids
PGC-1α: Peroxisome Proliferator-Activated Receptor Gamma Coactivator-1 Alpha
PHO: Primary Hypertrophic Osteoarthropathy
PID: Primary Immunodeficiency
PI-4P: Phosphatidylinositol 4-Phosphate
PAMP: Pathogen-Associated Molecular Pattern
PPI: Proton Pump Inhibitor
PRC2: Polycomb Repressive Complex 2
PRR: Pattern Recognition Receptor
ROS: Reactive Oxygen Species
RIPK1: Receptor-Interacting Serine/Threonine-Protein Kinase 1
RNF186: Ring Finger Protein 186
SAM: S-Adenosylmethionine
SCFA: Short-Chain Fatty Acid
scRNA-seq: Single-Cell RNA Sequencing
SCD: Specific Carbohydrate Diet
SMARCA4: SWI/SNF-Related Matrix-Associated Actin-Dependent Regulator of Chromatin Subfamily A Member 4
SMARCA5: SWI/SNF-Related Matrix-Associated Actin-Dependent Regulator of Chromatin Subfamily A Member 5
SOCS3: Suppressor of Cytokine Signaling 3
STAT: Signal Transducer and Activator of Transcription
Th17: T Helper 17 Cell
TET: Ten-Eleven Translocation Enzyme
TIFAB: TRAF-Interacting Protein with Forkhead-Associated Domain B
TLR: Toll-Like Receptor
TNF: Tumor Necrosis Factor
Treg: Regulatory T Cell
TTC7A: Tetratricopeptide Repeat Domain 7A
UC: Ulcerative Colitis
VEO-IBD: Very Early-Onset Inflammatory Bowel Disease
WES: Whole-Exome Sequencing
XIAP: X-Linked Inhibitor of Apoptosis Protein
